# Reducing Transversus Abdominis Release Hernioplasty Operative Volume Using Preoperative Botulinum Toxin Injections

**DOI:** 10.7759/cureus.94178

**Published:** 2025-10-09

**Authors:** Maxim A Chinikov, Hassan Othman, Razmik A Keshishyan, Malik K Al-Ariki, Hasan Saghir, Abubakar I Sidik

**Affiliations:** 1 Department of Hospital Surgery, Peoples’ Friendship University of Russia, Moscow, RUS; 2 Department of Surgery, Moscow State Budgetary Institution of Healthcare “L.A. Vorokhobov City Clinical Hospital No. 67”, Moscow, RUS; 3 Department of Surgery, Patrice Lumumba Peoples’ Friendship University of Russia, Moscow, RUS; 4 Department of Cardiothoracic Surgery, Peoples’ Friendship University of Russia, Moscow, RUS; 5 Medical School, Novosibirsk State Medical University, Novosibirsk, RUS; 6 Department of Cardiovascular Surgery, Peoples’ Friendship University of Russia, Moscow, RUS

**Keywords:** abdominal wall reconstruction, botulinum toxin type a, postoperative ventral hernia, transversus abdominis release, transversus abdominis release hernioplasty, ventral hernia

## Abstract

Large postoperative ventral hernias present significant challenges for repair, particularly in patients with comorbidities and wide defects. Here, we report the case of a 65-year-old male with a recurrent midline postoperative ventral hernia classified as M1-4W3R1 according to the European Hernia Society. CT revealed a defect measuring 15.1 × 18.2 cm with loss of domain. To facilitate closure, the patient underwent preoperative intramuscular injections of 500 units of botulinum toxin type A (BTA) into the external oblique, internal oblique, and transversus abdominis muscles under ultrasound guidance. Four weeks later, imaging demonstrated elongation of the lateral abdominal muscles and reduction of the defect width to 10.8 cm, which allowed a change in operative strategy from bilateral to unilateral transversus abdominis release hernioplasty. Primary tension-free fascial closure was achieved, reinforced with a 30 × 30 cm retromuscular polypropylene mesh. The postoperative course was uneventful, and at the sixth-month follow-up, there was no recurrence, with a marked improvement in quality of life. This case illustrates the feasibility and effectiveness of preoperative BTA injections in reducing operative complexity and enabling safe repair of large recurrent ventral hernias.

## Introduction

Ventral hernias remain a challenging clinical condition, as lateral traction on the abdominal wall can enlarge the defect and make surgical repair more challenging [1]. For patients with large midline postoperative ventral hernias (POVHs), the standard operative approach is transversus abdominis release (TAR) hernioplasty [2-4]. The European Hernia Society (EHS) classification system is instrumental in characterizing these complex hernias, categorizing them by location (M), width (W), and recurrence (R), which aids in surgical planning and predicting complexity [5]. A key challenge in large hernias is loss of domain, a condition where a significant portion of the abdominal contents lies outside the abdominal cavity, leading to a reduction in functional intra-abdominal volume and contraction of the lateral muscles. To optimize outcomes in this high-risk group, several preoperative strategies have been introduced, including progressive pneumoperitoneum and injections of botulinum toxin type A (BTA) [6]. BTA, a potent neurotoxin, acts by chemically denervating the lateral abdominal wall muscles, leading to flaccid paralysis, muscle elongation, and a reduction in lateral tension [7,8]. The TAR procedure, a posterior component separation technique, allows for extensive medialization of the rectus complexes and wide mesh sublay placement in the retromuscular space [2,3]. These adjuncts promote tension-free fascial closure, reduce the risk of intra-abdominal hypertension, and lower the likelihood of postoperative complications or recurrence. The present case report illustrates the potential role of BTA in decreasing the extent of TAR hernioplasty required for the management of large POVHs.

## Case presentation

**Figure 1:**
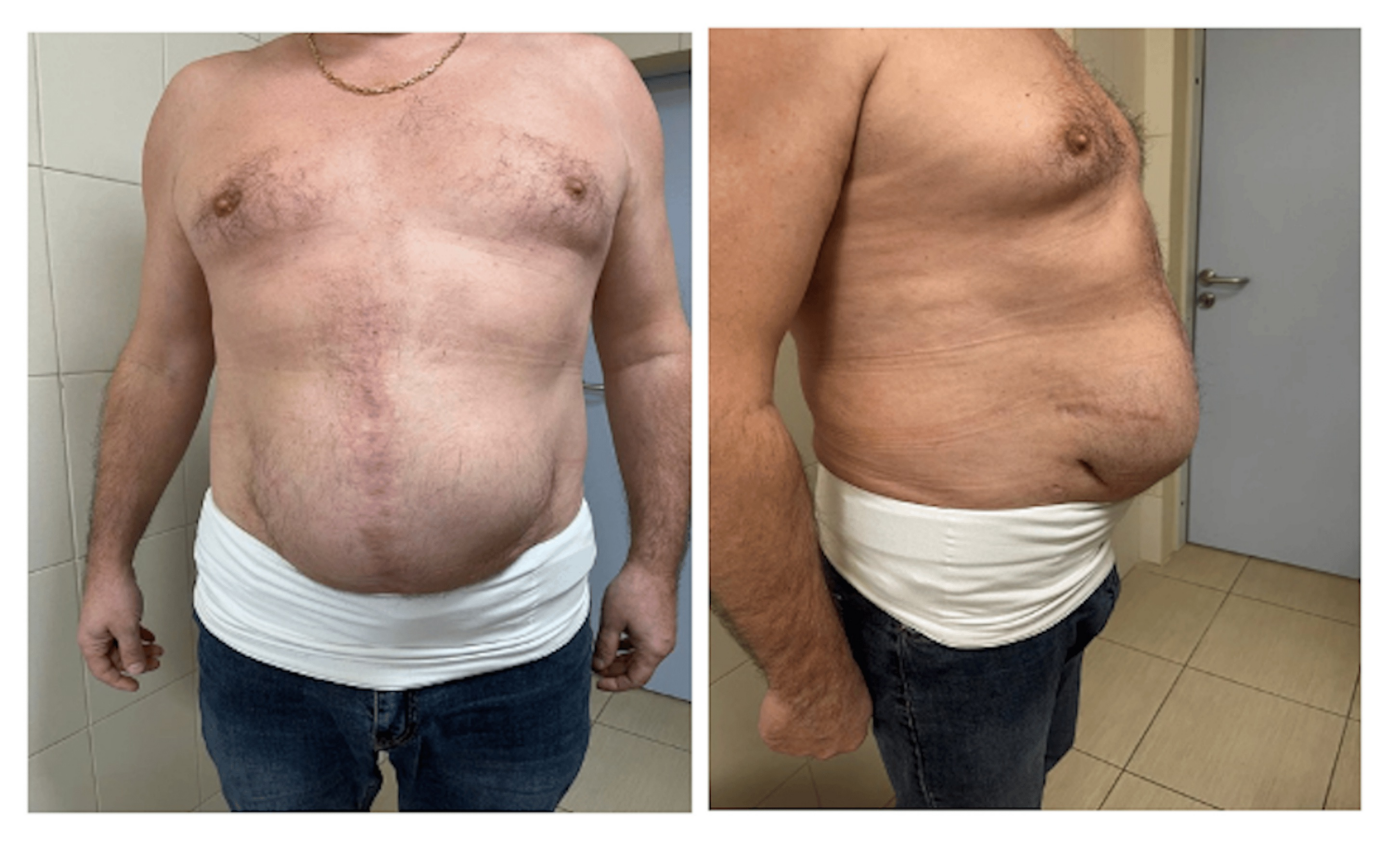
Physical examination revealing a 20 cm × 30 cm postoperative ventral hernial protrusion.

A 65-year-old man with a body mass index of 31.9 kg/m² presented in 2020 with a complaint of a midline hernial bulge at the site of a previous surgical scar. His surgical history included a laparotomy for Hartmann’s procedure in 2016, followed by a relaparotomy in 2018 for colostomy reversal with sigmoidorectal anastomosis. Two months after the latter operation, he developed a midline POVH and underwent autologous tissue repair during the same year. His medical history was significant for coronary artery disease, exertional angina (functional class II), post-infarction cardiosclerosis after myocardial infarction in 2020, and diffuse toxic goiter. On examination, a large, reducible midline hernia measuring approximately 20 × 30 cm was noted, which could be manually repositioned into the abdominal cavity. The cough impulse test was positive (Figure 1).

**Figure 2:**
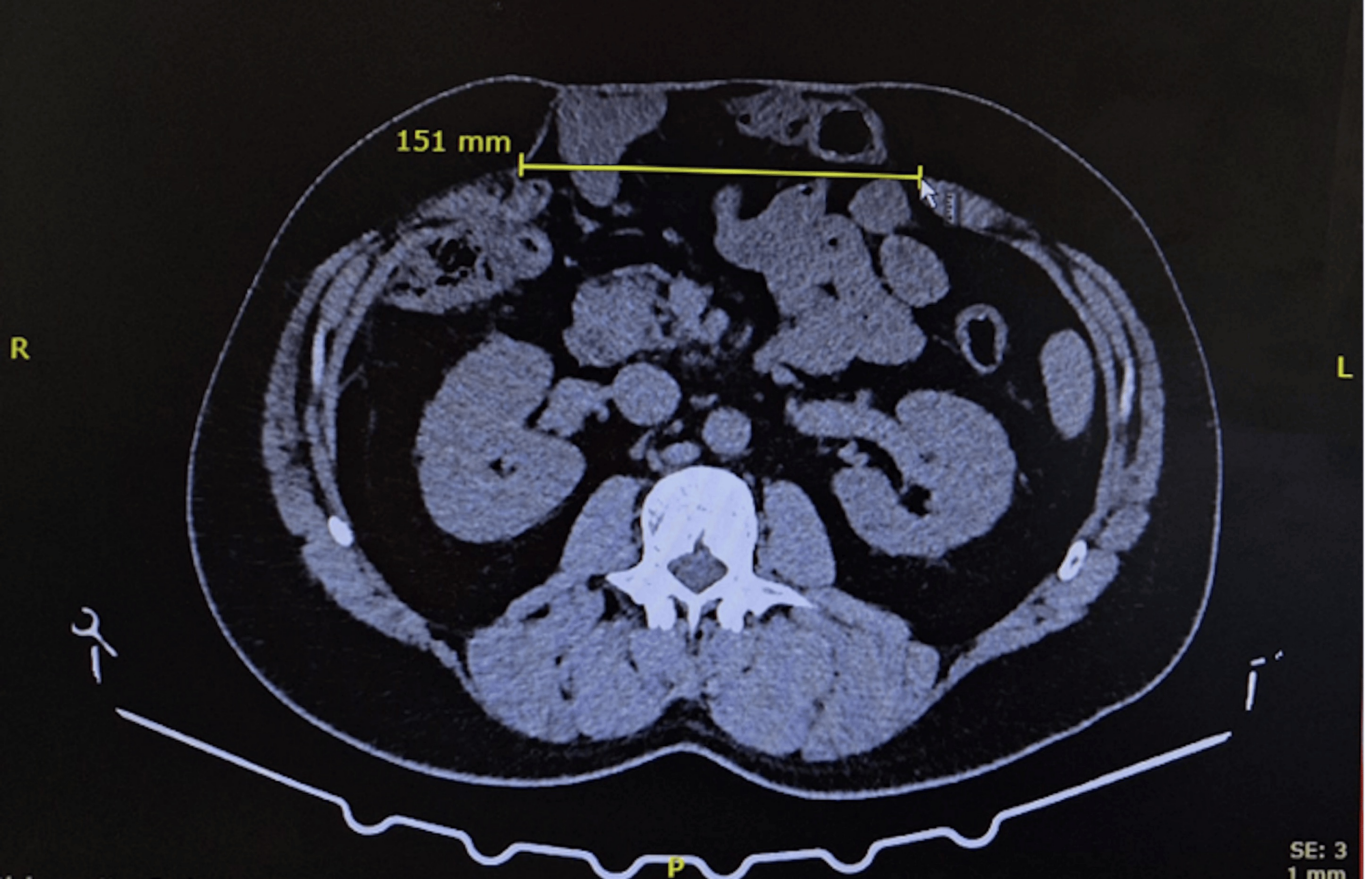
Abdominal CT scan of showing a 15.1 cm × 18.2 cm aponeurotic defect. The image shows the hernial orifice with a maximum width of 15.1 cm in the mesogastric region. A hernial sac containing loops of the small intestine and adipose tissue is also identified.

Abdominal CT (Figure 2) demonstrated a large POVH with an aponeurotic defect measuring 15.1 × 18.2 cm. The transverse diameter of the hernia defect (15.1 cm) was measured on axial CT slices as the maximum distance between the fused aponeurotic edges at the midline. The craniocaudal extent (18.2 cm) was measured on sagittal reconstructions. The hernial sac contained small bowel loops along with a portion of the large intestine, and features consistent with loss of domain were present. No additional significant abnormalities were detected. The Tanaka index was calculated as 0.22. The Sabbagh index was 18%.

Based on clinical assessment and CT findings, the patient was diagnosed with a recurrent (R1) midline subxiphoid (M1) POVH with a defect exceeding 10 cm in width (W3), corresponding to M1-4W3R1 under the EHS classification [5]. According to EHS guidelines, the recommended management for such cases is a component separation technique [2-4]. However, in view of the patient’s comorbidities and the need to minimize operative extent, a staged approach was chosen: initial preoperative BTA injection followed by definitive surgical repair.

**Figure 3:**
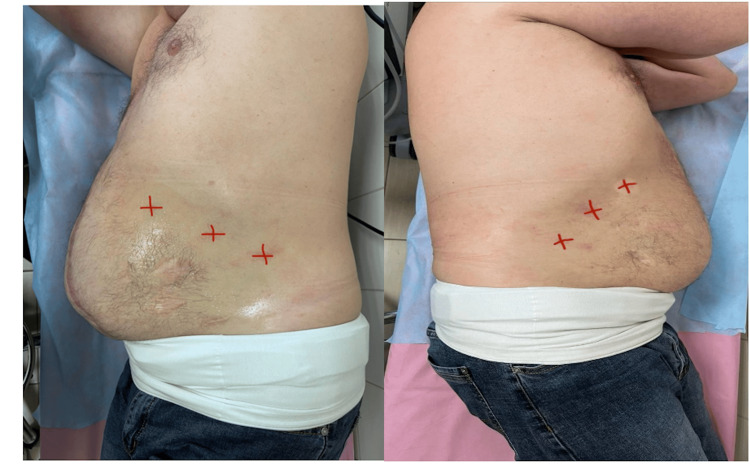
Botulinum toxin type A injection (injection sites marked with a red marker).

For preoperative optimization, and after confirming the absence of allergy, a total of 500 units of BTA (Dysport) were injected into the lateral abdominal wall muscles under ultrasound guidance. The procedure was performed aseptically with local anesthesia (2% lidocaine). Injections were delivered at three sites per side, targeting the external oblique, internal oblique, and transversus abdominis muscles with a total of 6 depots (83.3 units per depot), distributed evenly along the mid-axillary line (Figure 3).

**Figure 4:**
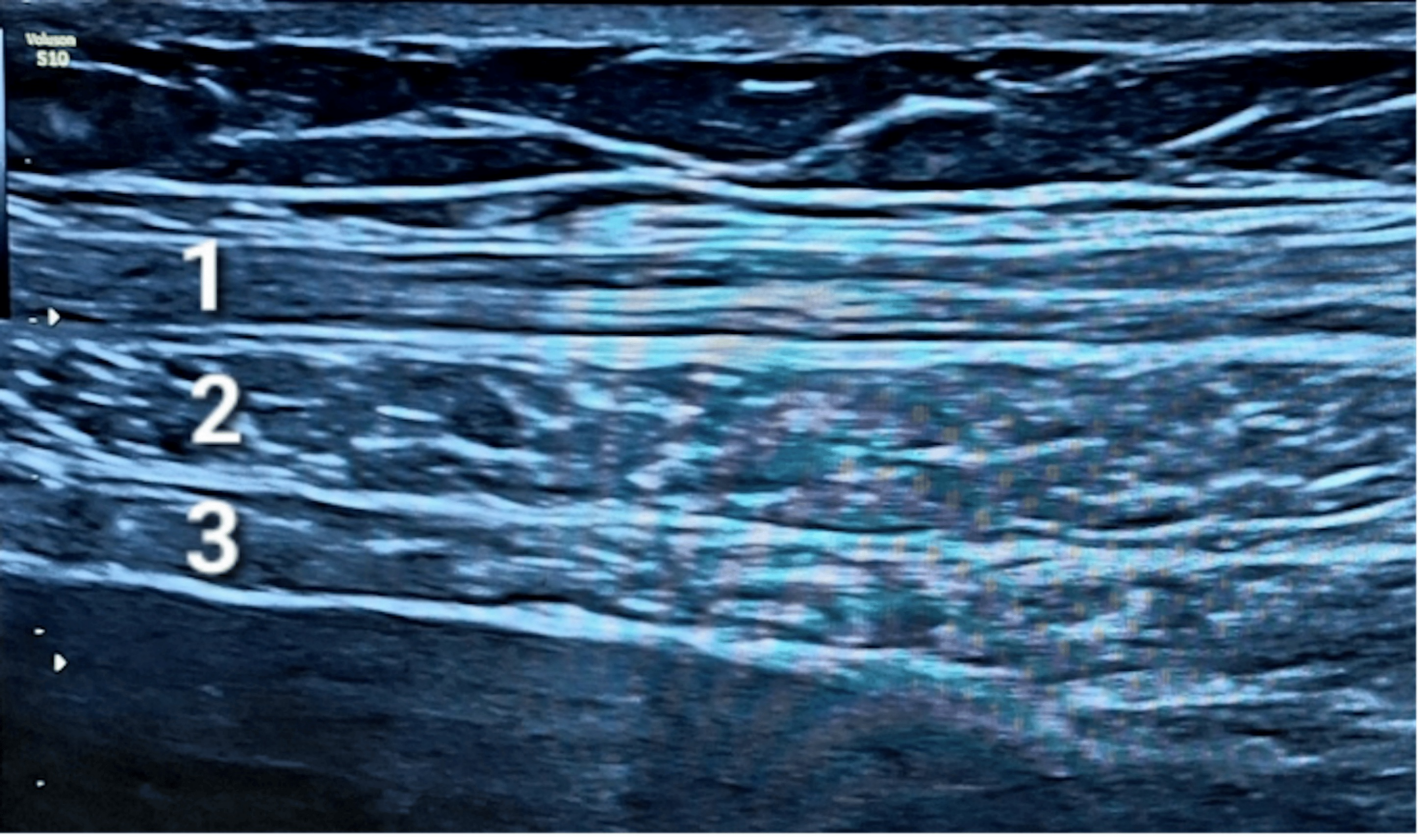
Abdominal wall ultrasound scan after intramuscular botulinum toxin type A injection. (1) External oblique muscle. (2) Internal oblique muscle. (3) Transversus abdominis muscle.

Before administration, 500 units of the botulinum toxin preparation were diluted in 60 mL of physiological saline. The resulting solution was injected at 10 mL per injection site, corresponding to 3 mL per muscle (Figure 4).

**Figure 5:**
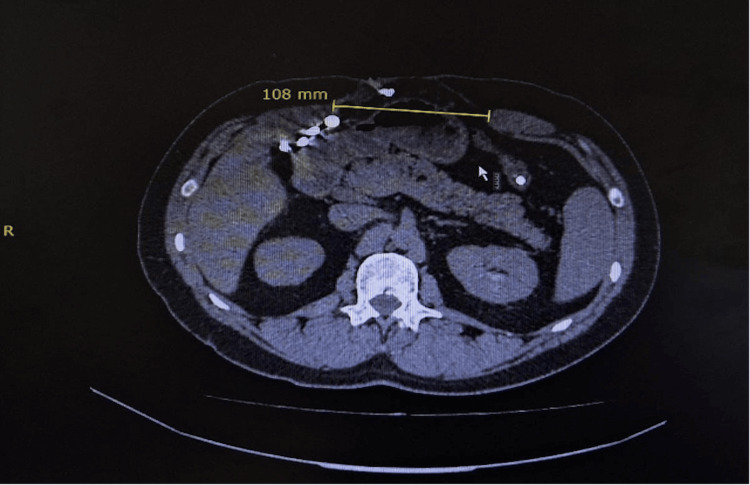
Abdominal CT scan four weeks after botulinum toxin type A administration revealing a reduction in the hernia transverse size to 10.8 cm.

The BTA injections were completed without complications, and the patient was discharged for outpatient monitoring with instructions to use an abdominal binder and undergo a repeat abdominal CT scan after four weeks to guide surgical planning. At the fourth-week follow-up, clinical assessment showed relaxation of the lateral abdominal wall muscles and a decrease in the transverse hernia defect to 11 cm. The repeat CT scan confirmed these changes in the abdominal wall musculature (Figure 5). The follow-up CT scan (Figure 5) showed elongation of the lateral abdominal muscles by 2.15 cm on each side (a total increase of 4.3 cm), along with a reduction of the hernia defect’s transverse diameter to 10.8 cm. These findings indicated that preoperative BTA injections effectively decreased the maximal width of the hernial orifice, allowing the operative plan to be adjusted to a unilateral TAR hernioplasty. The patient’s overall condition was assessed as American Society of Anesthesiologists class III.

**Figure 6:**
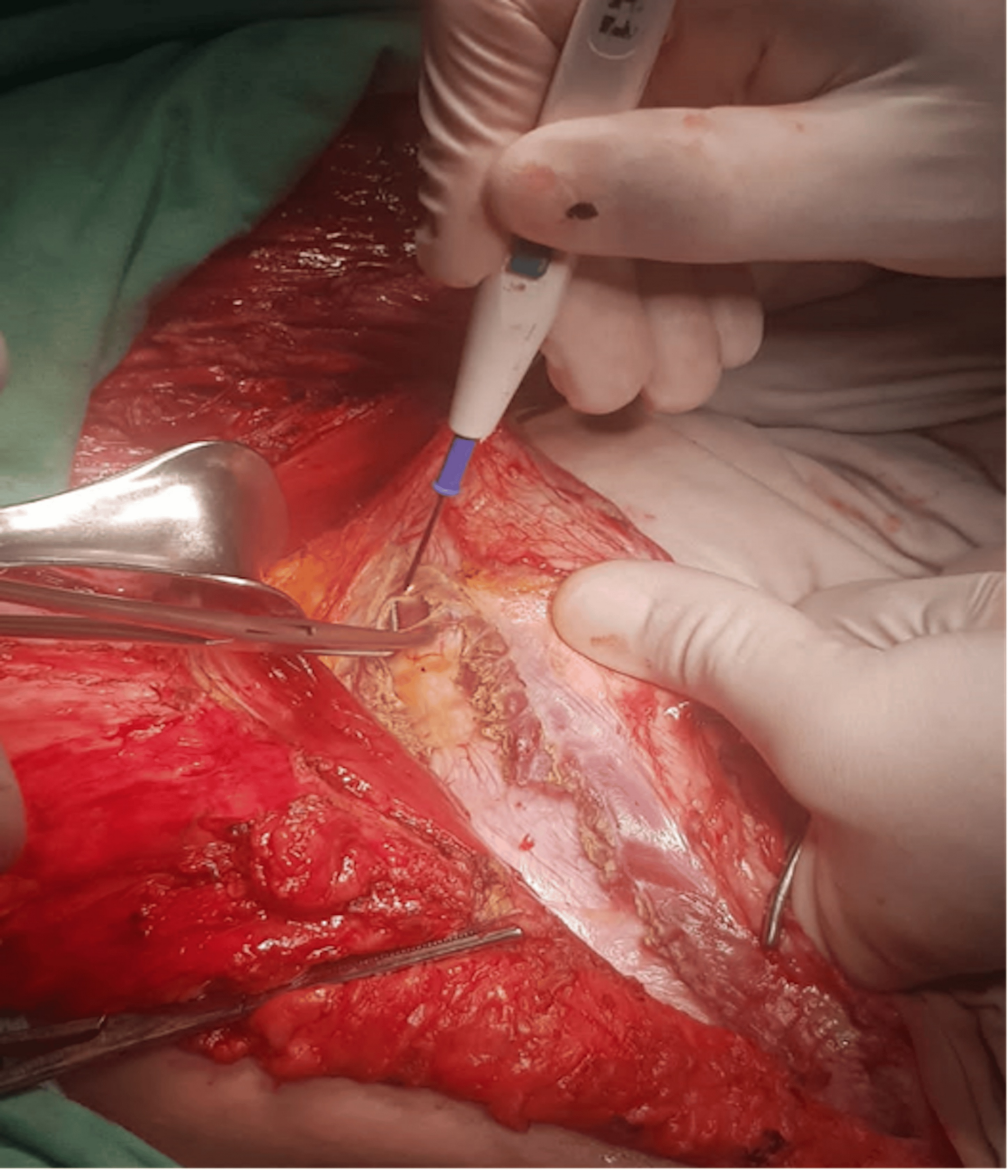
Surgical release of the right transversus abdominis muscle insertion.

Surgery was performed under endotracheal anesthesia. After routine preparation of the operative field, herniolaparotomy with excision of the old midline scar was undertaken. Bilateral dissection of the retromuscular space was then performed. Once the perforating vessels were identified, the fibers of the right transversus abdominis muscle were divided (Figure 6). Medialization of the posterior rectus sheath laminae allowed for tension-free closure of the midline defect, achieved with a continuous 1-0 polypropylene suture while maintaining an intraoperative intra-abdominal pressure (IAP) of 10 mmHg, measured via an indwelling urinary catheter connected to a pressure transducer. A 30 × 30 cm polypropylene mesh was then positioned over the posterior rectus sheath laminae and secured to the aponeurotic structures with interrupted sutures. The retromuscular space was drained, and the wound was closed in layers. The procedure lasted 2 hours and 45 minutes, and no intraoperative complications were noted.

**Figure 7:**
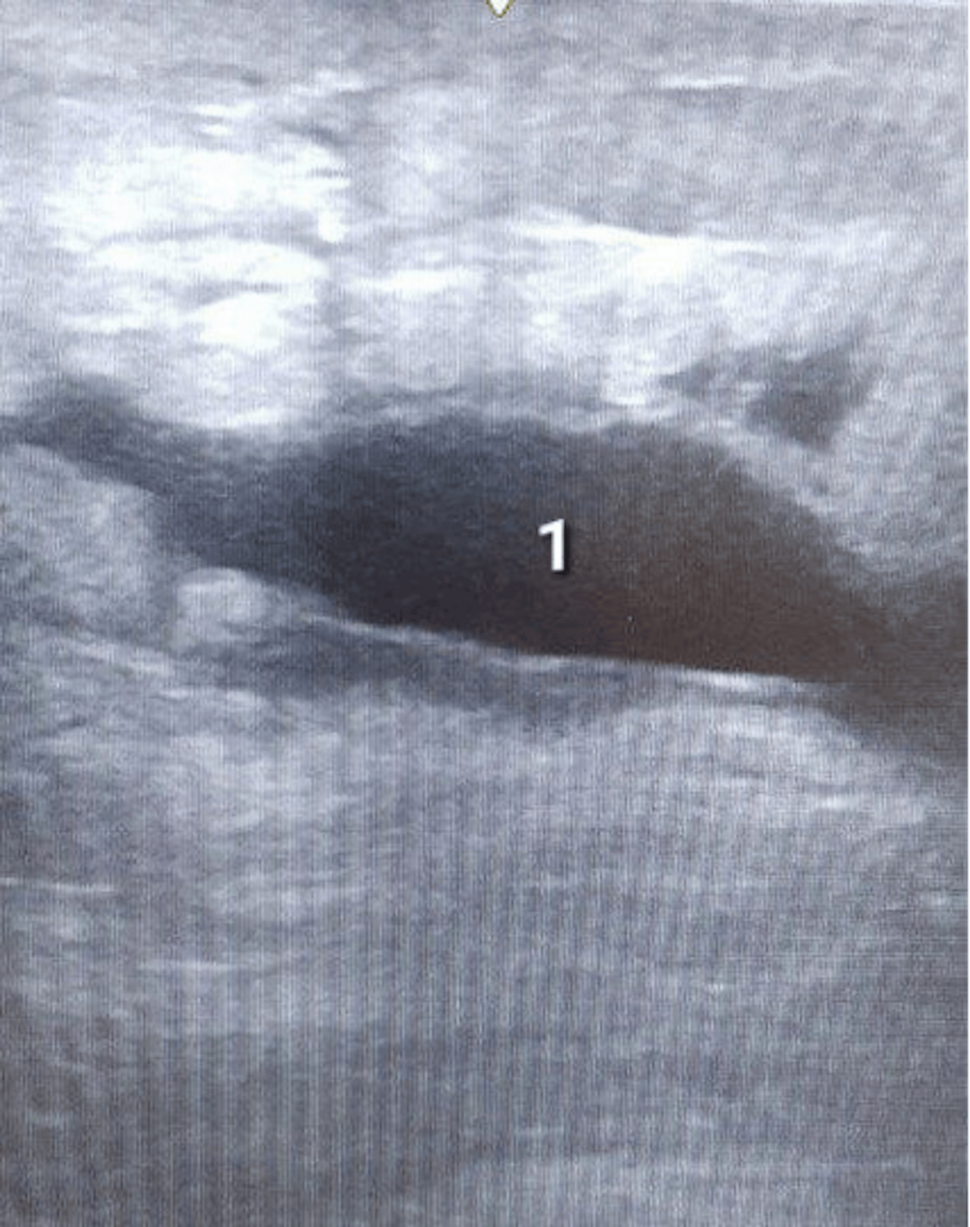
Abdominal wall ultrasound on postoperative day four showing hypoechoic fluid collection (exudate) in the subcutaneous adipose tissue.

Postoperatively, the patient remained stable with no complications during the first 24 hours (IAP 5 mmHg). However, on postoperative day four, an ultrasound of the anterior abdominal wall identified a localized fluid collection within the subcutaneous tissue (Figure 7).

**Figure 8:**
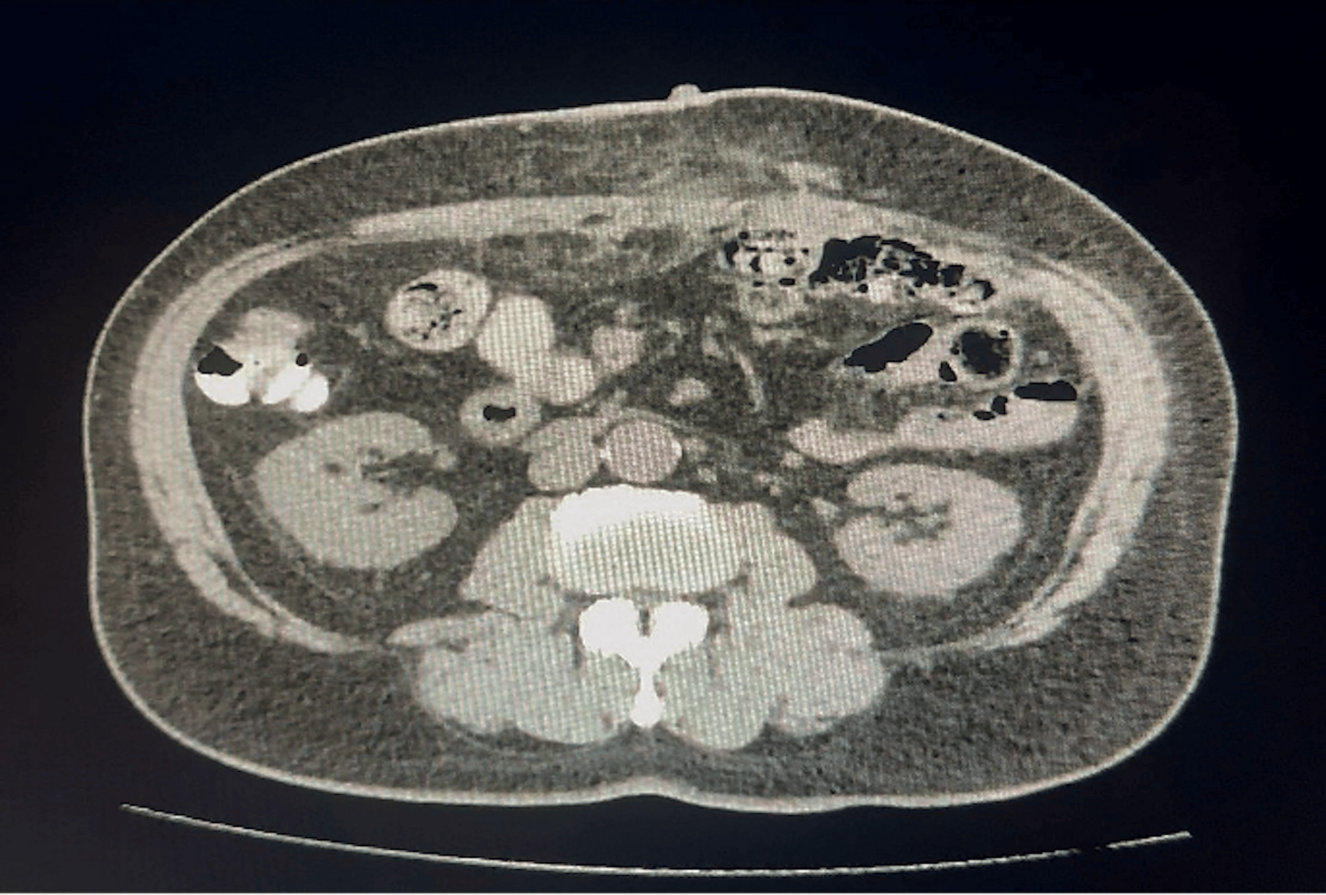
Abdominal CT scan at one month postoperatively.

The fluid collection was aspirated under ultrasound guidance, yielding 20 mL of serosanguinous fluid. The remainder of the postoperative course was uneventful. The patient was discharged on postoperative day five in a stable condition, with a total hospital stay of five days, and skin sutures were removed on day 10. Figure 8 shows an abdominal CT scan obtained one month after the procedure.

At the 12-month follow-up, there were no indications of hernia recurrence or abdominal wall asymmetry. Clinical examination confirmed the absence of recurrence or bulging. Quality of life assessment with the EuroQol 5-Dimension 5-Level questionnaire demonstrated marked improvement, with the patient’s score increasing from 30 points preoperatively to 85 points postoperatively and maintained at this level at 12 months.

## Discussion

At four weeks after BTA administration, marked morphofunctional changes were evident in the anterior abdominal wall, including elongation of the lateral muscles, narrowing of the hernia defect, and an increase in abdominal cavity volume. These effects created favorable conditions to perform a unilateral rather than a bilateral TAR hernioplasty, while still achieving complete primary tension-free fascial closure. Importantly, the patient did not demonstrate a loss of domain condition.

The postoperative course was uneventful, with no major complications, further supporting the safety and feasibility of BTA in the preoperative optimization of patients with large midline hernias, particularly by reducing the risk of intra-abdominal hypertension. Most reports in the literature describe BTA use in combination with anterior component separation for this patient group [7,8]. This case presents an initial experience with preoperative BTA injection followed by unilateral TAR hernioplasty, and further investigation is warranted to validate this approach.

Ventral hernias represent a frequent postoperative complication, particularly following midline laparotomy, with incidence rates exceeding 30% [9]. The management of patients with large midline hernias (W3 according to the ESH classification) presents particular challenges. The gold-standard treatment for this patient category involves various modifications of component separation techniques, i.e., anterior separation (Carbonell modification) or posterior separation (TAR procedure). Currently, most surgeons favor TAR as the preferred option for treating large midline ventral hernias [10-12].

Surgical practice encounters clinical situations requiring unilateral component separation. The main indications for this approach include lateral and parastomal hernias, as well as cases requiring the creation of a protective barrier between mesh implants and internal organs [13-15]. However, it should be noted that these indications remain subject to active discussion among specialists, and the final decision regarding the type of repair is often made intraoperatively based on individual patient anatomical characteristics.

Another crucial consideration in selecting component separation techniques is the hernia recurrence rates. According to Wegdam et al., the recurrence rate two years after TAR procedure was 4%, significantly lower than after anterior component separation (13%) [16]. According to Christopher et al., the average time to recurrence after the TAR procedure was 17 months [17], while Rabie et al. reported 13 months [18], and Oprea et al. indicated recurrence occurrence within 5-11 months (median = 8 ± 3 months) [19].

The application of robotic technologies in treating large midline ventral hernias aims to reduce postoperative complications and recurrence rates, ultimately improving patient quality of life. In the study by Nguyen et al., outcomes of 200 robotic-assisted TAR procedures were evaluated [20]. The authors reported postoperative complications in only 1% of patients (trocar-site infection in one patient and mesh migration in one patient). Hernia recurrences were recorded in four (2%) patients, predominantly in the group with large (115 cm²) and complex hernia defects. Researchers note that despite the existing learning curve, the technique demonstrates clear clinical advantages, and operative duration decreases with increasing surgical experience.

According to Kolygin et al., no hernia recurrences were observed following robotic-assisted TAR procedures in 17 patients [21]. Patient follow-up periods ranged from three months to three years. In a large-scale cohort study by Fry et al., treatment outcomes of 161,415 patients were analyzed [22]. The recurrence rate for robotic TAR was 13.43% and did not differ significantly from laparoscopic (12.33%) or open (12.74%) approaches. This may be associated with technology implementation phases and the development of surgical skills.

In addition to various component separation techniques, surgeons managing large midline ventral hernias have an alternative method of chemical separation of lateral abdominal wall muscles using BTA, initially proposed by Cakmak et al. [23]. BTA injections into the lateral abdominal wall muscles inhibit acetylcholine release at neuronal synapses, leading to temporary muscle paralysis, increased muscle length, and facilitated closure of large hernia defects without risk of compartment syndrome. BTA application is indicated for W3 ventral hernias, both with and without loss of domain (Tanaka index >0.25) [24].

In certain clinical contexts, unilateral TAR is selected to limit the operative extent. Polyakov et al. suggested that its indications include combined midline and lateral hernias or the need to create a barrier between mesh and intra-abdominal organs [9]. Similarly, Riediger and Köckerling identified unilateral TAR as a useful approach for lateral and parastomal hernias [7], with comparable findings also reported by Vogel et al. [8].

Given the currently unclear criteria for unilateral TAR, the use of BTA in the preoperative period for POVH appears to be a promising adjunct [1-3]. By facilitating reduced operative volume, BTA may shorten surgical time and lower the risk of postoperative complications. Based on these findings, we continue to apply this strategy in comparable patients and expect that larger studies will help define clear indications for unilateral TAR in complex abdominal wall reconstruction.

## Conclusions

This treatment approach for large midline POVHs shows promise, as preoperative botulinum toxin administration provides chemical relaxation of the lateral abdominal muscles, allowing for a reduction in both operative time and the extent of TAR hernioplasty required.
